# Parental marital conflict and short video dependency: a moderated mediation model

**DOI:** 10.3389/fpsyg.2026.1742070

**Published:** 2026-01-29

**Authors:** Mengting Pan, Ting Xiao, Yanfei Wang, Yuling Yang, Yulu Liu, Yutao Chen, Yang Liu, Zhiru Liang, Bo Wang

**Affiliations:** 1 Southwest University, Chongqing, China; 2 School of Sport Science, Beijing Sport University, Beijing, China; 3 Department of Science and Education, Xilingol League Central Hospital, Xilinhot, Inner Mongolia, China; 4 College of Physical Education, Hunan Normal University, Changsha, China; 5 School of Sports Science, Jishou University, Jishou, China; 6 Department of Hepatobiliary Surgery, Xilingol League Central Hospital, Xilinhot, Inner Mongolia, China; 7 Baotou Medical College, Inner Mongolia University of Science and Technology, Baotou, Inner Mongolia, China

**Keywords:** parental marital conflict, short video dependency, stress, physical activity, adolescent

## Abstract

**Objective:**

This study aims to examine the mediating role of stress and the moderating effect of physical activity (PA) in the relationship between parental marital conflict (PMC) and adolescent short video dependency (SVD).

**Methods:**

This study employed convenience sampling to recruit 719 adolescents aged 12–18 years from northwestern Hunan Province, China (*M* = 14.22, SD = 1.46). The data encompassed PMC, adolescent SVD, stress levels, and PA. Analytical procedures—ranging from descriptive and correlational summaries to tests of indirect and conditional indirect effects—were executed in SPSS with the PROCESS macro.

**Results:**

After controlling for demographic covariates, PMC was significantly and positively associated with adolescent SVD and perceived stress. Mediation analyses further showed that stress significantly mediated the association between PMC and adolescent SVD. In addition, PA significantly and negatively moderated the stress–SVD link, such that the positive association between stress and adolescent SVD was weaker among adolescents with higher levels of PA.

**Conclusion:**

These findings clarify how PMC translates into adolescent SVD at the psychological level. Stress was identified as a mediating factor, while PA, as a moderating mechanism, may attenuate the relationship between stress and adolescent SVD.

## 1 Background

The internet, as a global communication technology, has significantly transformed human interaction, work patterns, and daily life. However, despite its many benefits, it is also associated with several negative consequences, such as excessive or problematic internet use (Andreassen and Pallesen, 2014; Casale, 2020). This behavior is commonly referred to as “internet addiction” in academic discourse (Shaw and Black, 2008). Although the *Diagnostic and Statistical Manual of Mental Disorders* (DSM-5) (2013) does not classify internet addiction as an official diagnostic category or health issue, research suggests that excessive internet use can harm both physical and mental well-being (Valkenburg et al., 2022). As a result, the concept of internet addiction has been widely adopted in academic literature (Te, 2018; Weinstein and Lejoyeux, 2010). As the internet matures, social media ecosystems have proliferated into ever more varied niches, with short videos emerging as a prominent trend. These platforms, characterized by highly engaging, fast-paced, and personalized content, have increasingly occupied people’s leisure time (Zhang et al., 2019). The excessive use of short videos has attracted significant academic attention (Akhtar et al., 2024; Ye et al., 2022). Given the striking similarity between excessive use of short videos and traditional forms of internet addiction, the term “short video dependency” has been coined. The term denotes a pattern of short-video engagement that becomes compulsive—eliciting adverse outcomes when use persists and intrusive craving when access is removed (Griffiths, 2012; Hou et al., 2019). Adolescents now constitute the core user base of short-video platforms (CINIC, 2023a). Evidence shows that deepening adolescent immersion in short-video platforms is compromising their psychological well-being, physical health, and day-to-day functioning (Wang et al., 2025). This includes issues such as memory loss and failures (Xu et al., 2023), Affective distress—manifested as depressive mood and heightened stress (Sha and Dong, 2021), negative emotional states such as stress and depression (Zhu et al., 2024), social interaction difficulties (Zhang, 2023), cognitive and behavioral problems (Xu et al., 2023), and changes in family dynamics. Given its far-reaching influence on youth, unpacking the mechanisms behind short-video effects is now imperative.

### 1.1 Parental marital conflict, short video dependency

As a primary layer of the educational ecology, the family exerts enduring influence over adolescents’ personality formation, value systems, and behavioral routines (Shen, 2023). Within the family context, positive relational dynamics between parents are essential for maintaining family harmony and cohesion (Grevenstein et al., 2019). However, parental marital conflict (PMC) can negatively impact adolescents’ cognitive and emotional development (Yang et al., 2025). PMC refers to any disagreements within the family that affect both physiological and psychological aspects, and it serves as a key predictor of family cohesion and overall life quality (Cummings and Davies, 2002; Erel and Burman, 1995). According to the Emotional Security Theory (Cummings and Miller-Graff, 2015), frequent and intense parental conflict can undermine the emotional security of children and adolescents. When marital disputes result in negative emotional outcomes for adolescents (McNeil and Zeman, 2021), short videos, as a readily accessible source of entertainment and emotional relief, may serve as a means of escaping these distressing emotions (Li et al., 2021). Furthermore, research suggests that negative parental behaviors within the family environment can contribute to the development of psychological symptoms, such as anxiety, in adolescents (Kelly, 2000; Liu et al., 2024b), which, in turn, increases the likelihood of short video dependency (SVD). In sum, the study seeks to illuminate how PMC fuels adolescent SVD, providing valuable insights for clinical research and offering evidence-based recommendations for addressing SVD.

### 1.2 The mediating role of stress in the relationship between parental marital conflict and adolescent short video dependency

Parental relationship quality is pivotal to adolescents’ affective maturation and psychological adjustment (Bi et al., 2018). Youth reared amid persistent inter-parental strife routinely confront heightened emotional turbulence, with stress being the most common and significant manifestation (Chakraborty, 2023). Stress can be defined as the emotional and psychological strain induced by challenging or tense situations (Lecic-Tosevski et al., 2011). Empirical work consistently links PMC to elevated stress among adolescents (Lucas-Thompson et al., 2017). Specifically, conflicts and instability within the parental marriage often serve as triggers for stress responses in adolescents, leading to emotional turmoil and anxiety (Wahyuningsih et al., 2020). Further studies suggest that when adolescents experience stress resulting from marital conflict, they tend to seek alternative coping mechanisms to regulate their emotions (Liu et al., 2024b), with short video platforms emerging as a prevalent coping strategy. Studies have shown that stress is positively correlated with adolescents’ engagement in SVD. Furthermore, the level of stress has a significant positive correlation with the frequency of adolescents’ SVD (Hu and Huang, 2024; Liu et al., 2021; Mu et al., 2022). This phenomenon has also been reported in other regions (Donati et al., 2022; Journault et al., 2024; Stieger et al., 2023), suggesting that stress may be a key global factor influencing adolescent dependence on short videos. Therefore, enhancing the quality of marital relationships between parents and preventing mental health disorders are crucial for adolescents and may have significant benefits.

### 1.3 Physical activity regulates the relationship between stress and adolescent short video dependency

Physical activity (PA) is widely acknowledged to benefit both somatic and psychological well-being, has been shown to effectively mitigate excessive internet dependence and other forms of addictive behavior (Lin et al., 2020; Luo et al., 2024). Studies indicate that a key characteristic of SVD is the pleasure derived from consuming content (Chen et al., 2024), a process underpinned by dopaminergic signaling in mesolimbic and prefrontal circuits (Finlay and Zigmond, 1997; Yan et al., 2024). Dopamine is pivotal for both reward processing and stress reactivity (Pani et al., 2000). Prolonged exposure to stress can lead to dysregulation of the dopamine system (Baik, 2020; Stanwood, 2019), whereas PA has been demonstrated to regulate dopamine release (Gorrell et al., 2022). The pleasure derived from short videos plays a significant role in fostering dependence. Flow Theory posits that the dopaminergic surge accompanying intense concentration during PA fosters an immersive, flow-like state. This flow experience gratifies intrinsic pleasure-seeking, thereby weakening the pull of short-form video content (Nakamura and Csikszentmihalyi, 2009). This heightened flow experience can satisfy needs, thereby reducing SVD. Research has shown that moderate PA can help reduce individuals’ dependence on short videos (Luo et al., 2023; Zhang et al., 2022). Moreover, adolescents—juggling academic overload, ongoing neural maturation, and complex family dynamics—markedly escalate their short-video consumption (Wang et al., 2025; Xu et al., 2023). Although existing studies have confirmed the moderating effect of PA on SVA among adolescents, the underlying mechanisms linking stress and short-video dependence remain insufficiently explored.

Existing research indicates that examining demographic differences in core variables is a crucial prerequisite for ensuring the validity of studies (Fassett et al., 2022). In adolescent development research, factors such as gender, age, and education level may systematically influence perceptions of PMC, stress, and SVD (Chang, 2025; Cook et al., 2019; Osarenren, 2013). For example, girls tend to exhibit higher emotional sensitivity to marital conflict (Hosokawa and Katsura, 2019). Therefore, this study will prioritize examining group differences across key demographic variables—namely gender (male/female) and educational stage (middle school/high school)—with respect to PMC, stress, SVD, and PA. This approach will help ensure that subsequent mediation and moderation analyses are not confounded by potential demographic biases.

### 1.4 Overview

**Figure 1:**
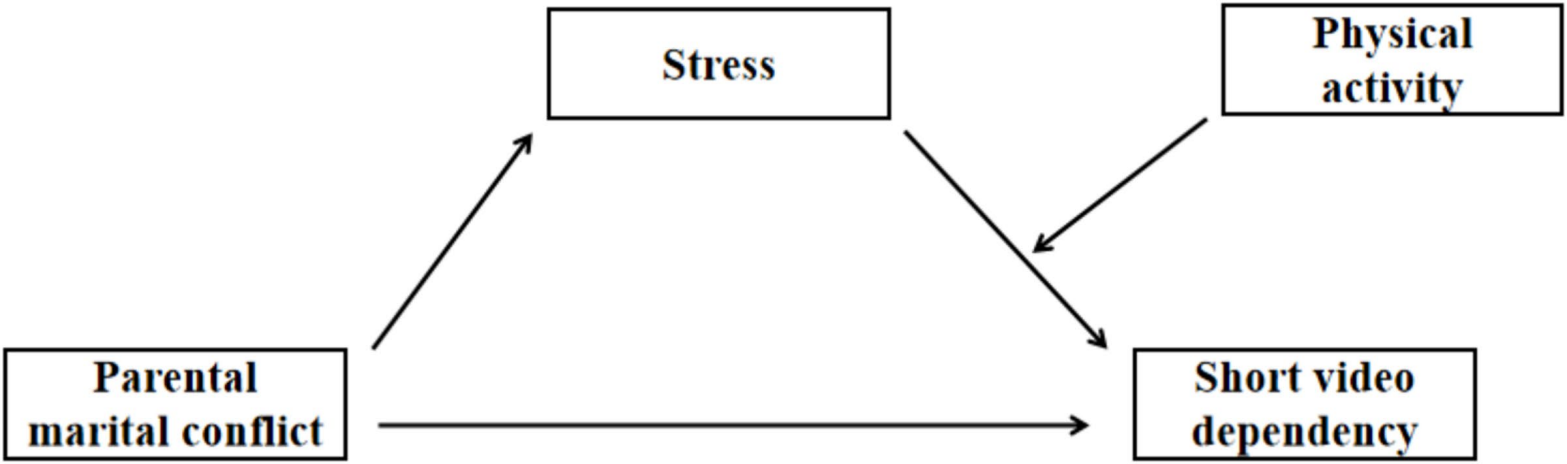
A moderated mediation hypothesized model.

Expanding prior insights, we interrogate how PMC translates into adolescent SVD, positing stress as mediator and PA as moderator of this pathway. Accordingly, we advance a moderated-mediation model (Figure 1) that situates stress between PMC and adolescent SVD, with PA attenuating or amplifying the indirect path. From this framework we derive the following hypotheses:

*H1*: There is a significant association between PMC and adolescent SVD.

*H2*: Stress mediates the relationship between PMC and adolescent SVD.

*H3*: PA significantly moderates the relationship between stress and adolescent SVD.

## 2 Methods

### 2.1 Participants

This study was conducted in September 2024 across two secondary schools in Hunan Province, utilizing a convenience sampling method to survey 756 adolescents. The questionnaire comprised measurement scales for PMC, adolescent SVD, PA, and anxiety levels. Prior to administering the survey, the research objectives were thoroughly explained, with an emphasis on the anonymity, confidentiality, and potential utility of the data. Participants were advised that they could withdraw at any point without penalty. The study was approved by the authors’ institutional Biomedical Ethics Committee, and informed assent/consent was obtained from adolescents and their guardians. After data collection, a rigorous screening process was employed to eliminate incomplete and patterned responses. A total of 719 valid questionnaires were returned (353 boys, 366 girls), with participant ages ranging from 12 to 18 years (*M* = 14.22, SD = 1.459).

### 2.2 Research methods

#### 2.2.1 Parental marital conflict

We employed the children’s perception of inter-parental conflict scale (Grych et al., 1992; Chi and Xin, 2003 revision) (Chi and Xin, 2003; Grych et al., 1992), was used in this study. The revised version of the scale consists of five items. One example item is: “Do you feel worried or scared when your parents argue?” The inventory uses a 4-point Likert format (1 = strongly disagree, 4 = strongly agree); summed scores range from 5 to 20, with higher values reflecting greater perceived conflict. This scale has been widely used in prior research in China on PMC (Liu et al., 2025; Zhao M. et al., 2024). Cronbach’s *α* = 0.78 in the present sample.

#### 2.2.2 Adolescent short video dependency

We administered Mao et al.’s (2022) 13-item Problematic Short Video Usage Scale, covering three core dimensions. One example item is: “Frequent or prolonged viewing of short videos often results in inadequate sleep or poor sleep quality.” Items are rated on a 5-point Likert continuum (1 = strongly disagree, 5 = strongly agree). The total score ranges from 13 to 65, with higher scores indicating a greater degree of problematic short video usage. This scale has been widely used in prior studies on Chinese adolescents and problematic short-video use (Mao and Jiang, 2023). The Cronbach’s alpha coefficient for the current sample is 0.852.

#### 2.2.3 Physical activity

Physical activity (PA) was indexed with Deqing’s (1994) revised scale, capturing intensity, duration and frequency in three subscores. The revised version includes three items. The composite PA index is computed as Intensity × (Duration – 1) × Frequency, yielding a 0–100 range. An example item on the scale is: “In the past month, how would you rate the intensity of your PA?” Higher scores reflect a greater amount of PA. The instrument has been extensively validated within Chinese adolescent populations (Liu et al., 2024c; Shen et al., 2024). In the present sample the scale yielded *α* = 0.61, a value considered acceptable for its intended purpose.

#### 2.2.4 Stress

The “Depression-Anxiety-Stress Scale (Short Version)” stress subscale, revised by Gong et al. (2010), was employed in this study. The seven-item subscale is rated on a 4-point Likert continuum (1 = strongly disagree, 4 = strongly agree). Higher scores denote elevated stress; This scale has been widely used in China (Sheng et al., 2023), internal consistency was good (α = 0.84).

### 2.3 Data analysis

Prior to hypothesis testing, common-method bias was evaluated. Following Podsakoff et al. (2003), a 40% variance threshold was adopted to flag appreciable common-method bias.

Subsequently, means and standard deviations of the four focal variables were obtained in SPSS 26.0, preceded by normality checks and followed by descriptive and correlational tests. Following Kim (2013) distributions were deemed approximately normal when |skewness| < 2 and |kurtosis| < 7. Normality checks satisfied parametric assumptions for all key variables. Independent samples *t*-tests and one-way analysis of variance (ANOVA) were used to examine differences across gender and grade levels, while Pearson correlation analysis was employed to assess the relationships between variables.

Furthermore, the PROCESS macro (version 4.0) in SPSS was used to test the mediation model. Before testing mediation, all variables were z-standardized. PMC was treated as the independent variable, adolescent SVD as the dependent variable, stress as the mediator, and PA as the moderator. Demographic covariates were entered into both the simple mediation (Model 4) and moderated-mediation (Model 14) analyses (Hayes, 2018). Due to the potential influence of demographic variables on the modeling process of this study, preliminary analyses and descriptions of these variables were conducted in the descriptive and correlational analysis sections. Significance was inferred from 95% bias-corrected confidence intervals obtained via 5,000 bootstrap draws, with effects deemed reliable when zero lay outside the interval (Berkovits et al., 2000). The significance level was set at 0.05.

## 3 Results

### 3.1 Common method deviation test

The common method bias test in this study was conducted using Harman’s single-factor test, which integrated the independent variable (PMC), dependent variable (SVD), mediating variable (Stress), and moderating variable (PA) for factor analysis. During the factor analysis, two factors with eigenvalues greater than 1 were identified. The first extracted factor explained 29.03% of the variance—well below the 40% cutoff—indicating negligible common-method bias.

### 3.2 Descriptive data

The study revealed significant gender differences in perceived PMC (*t* = −2.53, *p* < 0.05), stress (*t* = −6.436, *p* < 0.001), SVD (*t* = −3.192, *p* < 0.01), and PA (*t* = 6.36, *p* < 0.001). Specifically, male participants reported lower levels of PMC, stress, and SVD, whereas they exhibited higher levels of PA compared to female participants. Additionally, there were significant differences between grade levels in terms of SVD (*t* = −4.956, *p* < 0.001) and PA (*t* = −3.781, *p* < 0.001), with middle school students showing lower levels of SVD and PA compared to high school students (see Table 1).

**Table 1:** Difference analysis.

Variables		PMC		Stress		SVD		PA	
		Mean	sd	Mean	sd	Mean	sd	Mean	sd
Gender	Boys	9.68	3.26	12.75	4.179	33.99	10.42	22.64	23.92
	Girls	10.32	3.53	14.92	4.805	36.47	10.42	12.84	16.626
*t*		−2.53*		−6.436***		−3.192**		6.36***	
Grade	Junior high school	9.90	3.47	13.83	4.81	34.19	10.90	15.43	17.45
	Senior high school	10.29	3.24	13.91	4.13	38.11	8.68	23.69	28.09
*t*		−1.35		−0.219		−4.956***		−3.781***	

*p < 0.05; **p < 0.01; ***p < 0.001.

### 3.3 Correlational analyses

The results presented in Table 2 indicate that PMC is significantly positively correlated with SVD (*r* = 0.304, *p* < 0.001), stress (*r* = 0.498, *p* < 0.001). SVD also exhibits significant positive correlations with stress (*r* = 0.385, *p* < 0.001), as well as a significant negative correlation with PA (*r* = −0.138, *p* < 0.001). Stress shows a significant negative correlation with PA (*r* = −0.213, *p* < 0.01).

**Table 2:** Correlation analysis.

Variables	PMC	SVD	Stress	PA	Age
PMC	-				
SVD	0.304***	-			
Stress	0.498***	0.385***	-		
PA	−0.063	−0.138***	−0.213***	-	
Age	0.077*	0.205***	0.066	0.109**	-

*p < 0.05; **p < 0.01; ***p < 0.001.

### 3.4 Mediation model testing

**Figure 2:**
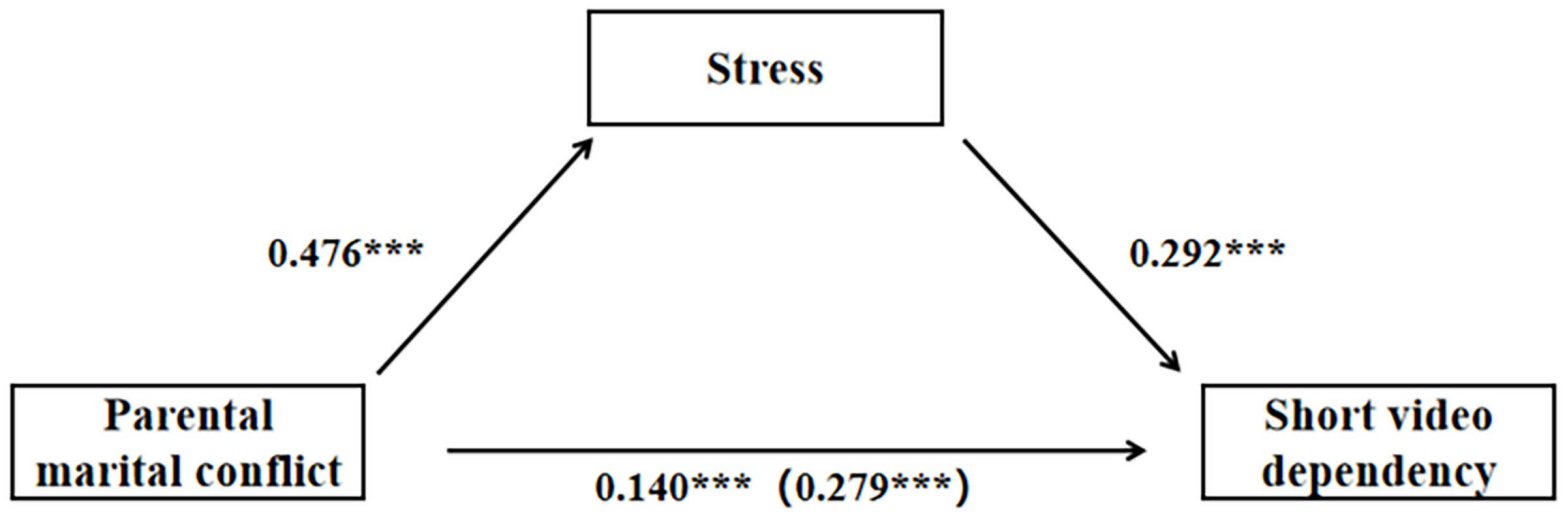
Test of the mediation model (***p < 0.001).

Based on the findings presented in Table 3 and Figure 2, a positive correlation was observed between PMC and adolescent SVD (*β* = 0.279, *p* < 0.001). Even after incorporating the mediator variable, stress, the positive relationship between PMC and adolescent SVD remained significant (*β* = 0.140, *p* < 0.001). Furthermore, a positive correlation was found between PMC and adolescent stress (*β* = 0.476, *p* < 0.001), as well as between stress and adolescent SVD (*β* = 0.292, *p* < 0.001). The proportion of the mediation effect is presented in Table 4.

**Table 3:** Tests the mediation model.

Variables	SVD			Stress			SVD		
	*β*	SE	*t*	*β*	SE	*t*	*β*	SE	*t*
PMC	0.279	0.035	7.964***	0.476	0.032	14.930***	0.140	0.039	3.619***
Stress							0.292	0.040	7.354***
Gender	0.107	0.035	3.034**	0.195	0.032	6.083***	0.050	0.035	1.431
Age	0.096	0.083	1.162	0.094	0.075	1.254	0.068	0.080	0.858
Grade	0.108	0.082	1.312	−0.052	0.075	−0.697	0.123	0.080	1.552
R^2^	0.138			0.287			0.199		
F	28.549***			71.922***			35.352***		

*p < 0.05; **p < 0.01; ***p < 0.001.

**Table 4:** Path analysis of mediation model.

Mediation model paths	Effect	SE	Bootstrap 95% CI	Proportion of mediating effect
Total effect	0.279	0.035	0.210, 0.348	
Direct effect	0.140	0.039	0.064, 0.216	
Indirect effect	0.139	0.024	0.094, 0.189	50.36%

### 3.5 Moderated mediation model testing

**Figure 3:**
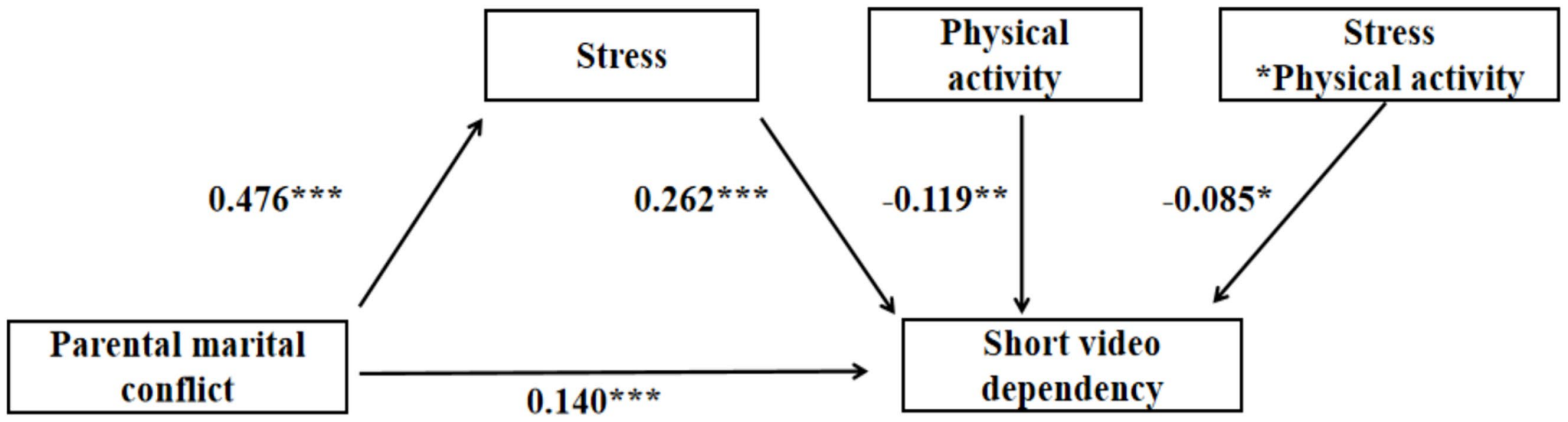
A moderated mediation model (*p < 0.05; **p < 0.01; ***p < 0.001).

**Figure 4:**
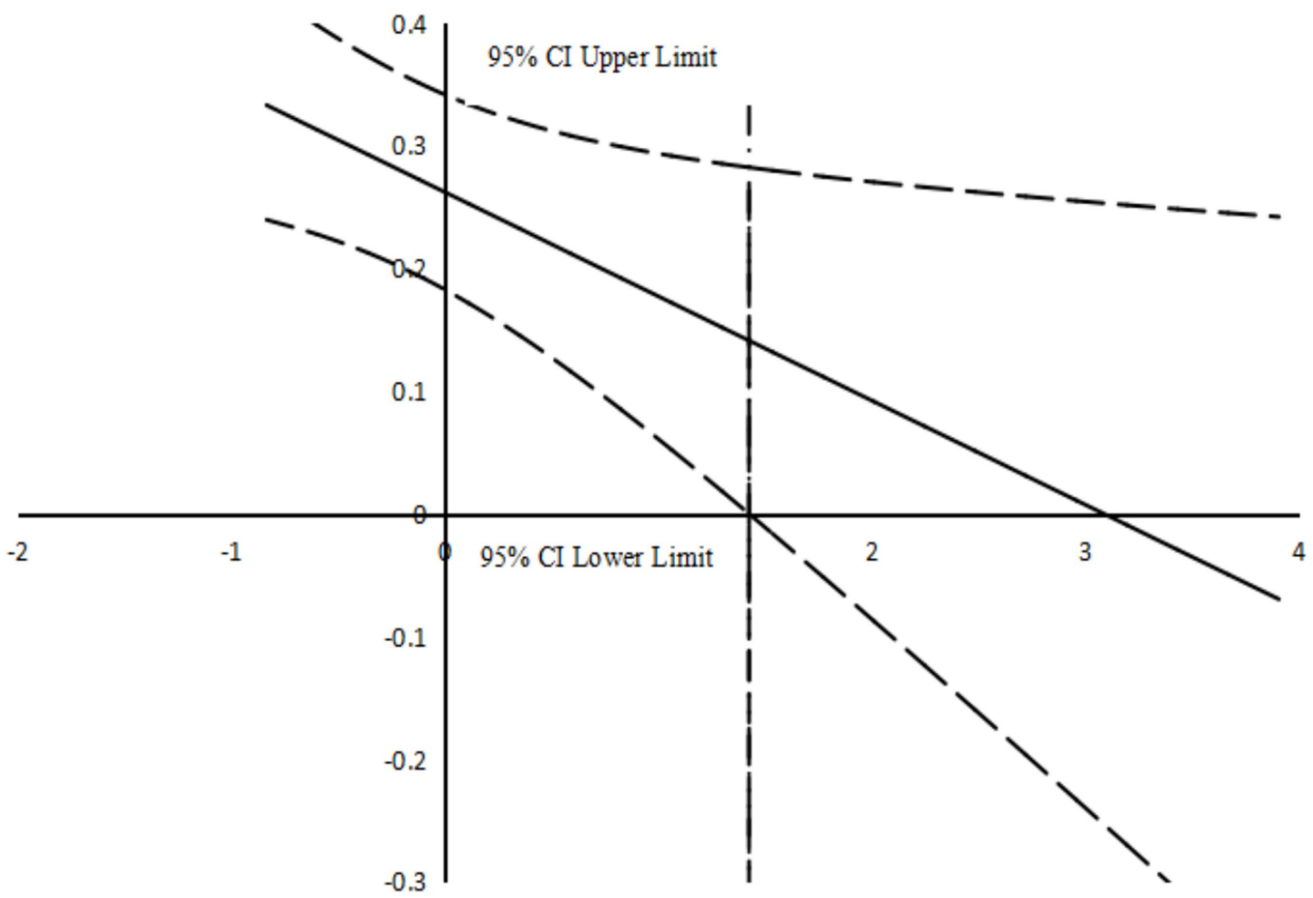
Johnson–Neyman method for analyzing the conditional effect of ‘Stress’ on ‘SVD.’ The Y-axis represents *β*-coefficients, and the *X*-axis represents levels of PA.

Prior to introducing the mediating variable, PMC was significantly associated with adolescents’ SVD (*β* = 0.279, *p* < 0.001). After the mediating variable was included in the model, PMC remained significantly associated with adolescents’ SVD, although the coefficient decreased (*β* = 0.140, *p* < 0.001). When demographic variables were controlled for and the moderating variable PA was incorporated, the results from Table 5 and Figures 3, 4 indicated that stress was positively correlated with adolescent SVD (*β* = 0.262, *p* < 0.001). Additionally, PA was negatively correlated with adolescent SVD (*β* = −0.119, *p* < 0.01). Moreover, the interaction between PA and stress was negatively correlated with adolescent SVD (*β* = −0.085, *p* < 0.05).

**Table 5:** Mediation-moderation test.

Variables	Stress			SVD		
	*β*	SE	*t*	*β*	SE	*t*
Gender	0.195	0.032	6.083***	0.032	0.035	0.911
Age	0.094	0.075	1.254	0.080	0.079	1.011
Grade	−0.052	0.075	−0.697	0.120	0.790	1.511
PMC	0.476	0.032	14.930***	0.140	0.039	3.742***
Stress (A)				0.262	0.040	6.472***
PA (B)				−0.119	0.038	−3.129**
A × B				−0.085	0.038	−2.246*
*R* ^2^	0.287			0.211		
*F*	71.922***			27.160***		

*p < 0.05; **p < 0.01; ***p < 0.001.

## 4 Discussion

This study explores the interrelationships among PMC, adolescent SVD, stress, and PA. PMC showed robust positive associations with both adolescent SVD and perceived stress. SVD aligned positively with stress yet negatively with PA, whereas stress and PA were inversely related. After adjusting for demographics and treating PA as a moderator, stress continued to significantly and positively predict adolescent SVD. Furthermore, PA was found to negatively predict adolescent SVD. The interaction between PA and stress also significantly predicted SVD among adolescents. These findings provide important theoretical and practical insights for research on adolescent mental and behavioral health.

Group differences in perceived PMC, stress, SVD, and PA were examined across gender and educational level using independent-samples *t*-tests. The results indicated that male participants reported significantly lower levels of perceived PMC, stress, and SVD compared to their female counterparts, while exhibiting significantly higher levels of PA engagement. Additionally, middle school students demonstrated significantly lower scores in SVD and PA compared to high school students. The observed gender differences may be attributed to the unique psychosocial developmental characteristics associated with puberty. Females tend to exhibit greater emotional sensitivity and responsiveness to family dynamics and emotional pressures, leading them to seek relief through short videos. In contrast, males show higher participation in PA, which may be influenced by societal expectations regarding male athletic performance and encouragement of physical capabilities during adolescence.(Gur and Gur, 2016; Lenroot and Giedd, 2010; Szadvári et al., 2023). With regard to educational level differences, high school students often face greater academic pressure, which may drive them to utilize short videos as a means of relaxation while also providing them with more opportunities to engage in PA (Liu and Lu, 2012; Steare et al., 2023). The results highlight the need for gender- and stage-specific mental-health interventions and educational initiatives that address the unique developmental demands of adolescents.

PMC emerged as a significant positive predictor of adolescent SVD; heightened conflict corresponded to increased dependency risk, aligning with prior evidence (Gao et al., 2018; Yang et al., 2016). Further analysis indicates that adolescents raised in households with frequent PMC tend to experience more severe psychological issues (Maya et al., 2024), which in turn leads them to seek psychological substitutes for emotional support. Short video platforms have increasingly become the psychological dependency target for these adolescents. Excessive use of short video platforms may eventually lead to SVD (Ye et al., 2025). Additionally, the study found that adolescents from families with frequent marital conflict often experience lower levels of emotional care and family support (Kelly, 1998). This lack of emotional connection further exacerbates their dependency on short video platforms. Accordingly, Hypothesis 1 is supported: Parental marital conflict forecasts elevated adolescent short video dependency.

We next examined whether stress transmits the influence of PMC to adolescent SVD. Persistent inter-parental conflict repeatedly exposes adolescents to negative affect, fostering sustained psychological distress (Wahyuningsih et al., 2020). When adolescents fail to effectively cope with these emotions, they are more likely to exhibit stress symptoms. Family-systems perspectives (Watson, 2012), regard PMC as a primary stressor that propagates affective strain and behavioral reactions throughout the household, with adolescents especially vulnerable to such systemic tension (Lucas-Thompson and Goldberg, 2011). Amid the swift ascent of short-form video platforms in modern society, emotionally distressed adolescents often seek solace in the utopian world created by these platforms as a way to escape from real-world pressures (Liu et al., 2024b). However, this escapism does not address the underlying emotional turmoil and may, in fact, contribute to the intensification of SVD. Longitudinal work has established a bidirectional link between stress and SVD: elevated stress corresponds to heavier consumption, while prolonged use, in turn, intensifies stress (Hu and Huang, 2024). Collectively, the evidence outlined above supports Hypothesis 2: stress functions as a mediator linking PMC to adolescent short video dependency.

Additionally, the findings underscore PA as a key moderator that attenuates the linkage between stress and adolescent SVD. Empirical work indicates that PA boosts adolescents’ self-esteem and body image while concurrently mitigating psychological stress and its associated symptoms (Di Liegro et al., 2019). This finding is consistent with social cognitive theory (Schunk and DiBenedetto, 2023), which posits that adolescents who observe peers or role models gaining physical health and social recognition (e.g., popularity as a member of a school sports team) through PA are more likely to imitate such behaviors (Atif et al., 2022), rather than becoming engrossed in short-form videos. Additionally, the enhancement of self-efficacy following the acquisition of sports skills can reduce the tendency to rely on short videos as an escapist coping mechanism (Zhao and Kou, 2024). Further evidence reveals an inverse association between PA and SVD, with greater engagement in PA corresponding to lower levels of SVD among adolescents (CINIC, 2023b; Liu et al., 2024a). Empirical analysis further confirmed the significant moderating effect of PA in the stress response process (Stults-Kolehmainen and Sinha, 2014; Zhao H. et al., 2024). As the level of PA increased, the mediating effect of stress gradually weakened, suggesting that PA may partially buffer the association between stress and adolescents’ SVD, which may be relevant to healthier growth and developmental outcomes in adolescence. Thus, Hypothesis 3 is supported.

PMC was found to foster adolescent SVD both directly and indirectly by intensifying stress-related symptomatology. Furthermore, PA emerges as an effective moderating mechanism, alleviating adolescents’ stress symptoms and consequently reducing their risk of SVD. In the process of developing SVD, parental conflict plays a crucial role in directly influencing the severity of this dependency. Therefore, it is imperative for parents to prioritize communication and cooperation, thereby fostering a supportive family environment conducive to the healthy development of adolescents. Additionally, engaging in PA offers adolescents a proactive strategy to cope with negative emotions, helping them better manage stress and regulate their emotional responses. This, in turn, reduces the risk of SVD and promotes overall psychological well-being. This study further explores the relationship between PMC and adolescent SVD, elucidating the underlying interaction mechanisms and providing a theoretical foundation for future research on the prevention and intervention of adolescent SVD. Future research could build on these findings by employing time-series analysis to investigate potential variables influencing adolescent SVD from the perspective of PMC, allowing for causal inferences to be drawn and thereby enhancing the effectiveness of SVD prevention strategies.

### 4.1 Advantages and limitations

A key contribution of this work is its simultaneous examination of stress as a mediator between PMC and adolescent SVD and of PA as a moderator of those pathways. Moreover, by foregrounding stress as a mediator and PA as a moderator, this study addresses a research gap that prior work has largely overlooked. Nevertheless, several limitations should be acknowledged. Firstly, a major limitation of this study is the use of a non-random sampling method and the failure to control for potential sources of bias, which may have affected the examination of demographic variables. Second, retrospective self-reports of PMC are vulnerable to recall bias, which may distort the obtained associations. Thirdly, due to the potential for various family-related issues, such as depression, domestic violence, and childhood abuse, to exert distinct negative effects on individuals, our study did not extensively investigate the nuanced impact of these variables on PMC. Future research could further explore the different dimensions of marital conflict and their specific consequences. Fourth, although the moderating effect observed in this study reached statistical significance, its magnitude was small and therefore warrants cautious interpretation in terms of practical relevance. In real-world settings, differences in PA levels alone may provide only limited buffering against stress. This pattern underscores the complexity of psychosocial and behavioral processes, in which the influence of any single factor is often modest. Future research should employ more sensitive and fine-grained measurements and examine potential synergistic effects between PA and other psychosocial resources to better explain when and for whom stress related SVD may be attenuated. In addition, an important limitation of this study is the omission of socioeconomic status (SES). SES is likely related to PMC, adolescents’ stress levels, and SVD; however, SES indicators were not collected in the present research (e.g., parental education, household income, family structure, or urban–rural residence). Consequently, residual confounding cannot be ruled out, and the observed associations may be overestimated. Future studies should incorporate SES-related measures to more rigorously evaluate the proposed mediation and moderation (moderated mediation) model. Lastly, the cross-sectional design limits causal inferences among the studied variables. Therefore, we recommend that future research adopt longitudinal designs to establish more robust causal inferences. Future research that remedies these constraints will refine understanding of how PMC shapes adolescent SVD through psychological processes.

## 5 Conclusion

This study investigates how PMC relates to adolescent SVD. The findings indicate that PMC is positively associated with adolescent SVD, with stress acting as a mediator in this relationship. Furthermore, PA buffers the association between marital conflict and short-video use. Using a moderated mediation model, this research provides a deeper understanding of the underlying mechanisms linking PMC and adolescent SVD. It also offers a novel perspective on the role of PA in promoting psychological well-being. The study suggests that parents should maintain effective communication and emotional management within their marriage to reduce negative psychological impacts on adolescents. Additionally, encouraging adolescents to engage in PA with their parents could strengthen parent–child relationships. Furthermore, parents should guide their children in using short videos in a balanced manner, encouraging them to select positive and healthy content to prevent excessive use. Supportive family climates and constructive parental communication mitigate the stress adolescents perceive from marital conflict.

## Data Availability

The raw data supporting the conclusions of this article will be made available by the authors, without undue reservation.
