# Correction: Parental marital conflict and short video dependency: a moderated mediation model

**DOI:** 10.3389/fpsyg.2026.1803048

**Published:** 2026-02-18

**Authors:** Mengting Pan, Ting Xiao, Yanfei Wang, Yuling Yang, Yulu Liu, Yutao Chen, Yang Liu, Zhiru Liang, Bo Wang

**Affiliations:** 1 Southwest University, Chongqing, China; 2 School of Sport Science, Beijing Sport University, Beijing, China; 3 Department of Science and Education, Xilingol League Central Hospital, Xilinhot, Inner Mongolia, China; 4 College of Physical Education, Hunan Normal University, Changsha, China; 5 School of Sports Science, Jishou University, Jishou, China; 6 Department of Hepatobiliary Surgery, Xilingol League Central Hospital, Xilinhot, Inner Mongolia, China; 7 Baotou Medical College, Inner Mongolia University of Science and Technology, Baotou, Inner Mongolia, China

**Keywords:** parental marital conflict, short video dependency, stress, physical activity, adolescent

Affiliation [3 Department of Science and Education, Xilingol League Central Hospital, Xilinhot, Inner Mongolia, China] was erroneously given as [6 Department of Hepatobiliary Surgery, Xilingol League Central Hospital, Xilinhot, Inner Mongolia, China].

The original version of this article has been updated.

